# Bromodomain Protein Inhibitors Reorganize the Chromatin of Synovial Fibroblasts

**DOI:** 10.3390/cells12081149

**Published:** 2023-04-13

**Authors:** Monika Krošel, Larissa Moser, Miranda Houtman, Jasna Friščić, Matija Tomšič, Oliver Distler, Markus H. Hoffmann, Caroline Ospelt, Kerstin Klein

**Affiliations:** 1 Center of Experimental Rheumatology, Department of Rheumatology, University Hospital Zurich, University of Zurich, 8091 Zurich, Switzerland; 2 Department of Rheumatology, University Medical Centre Ljubljana, 1000 Ljubljana, Slovenia; 3 Faculty of Medicine, University of Ljubljana, 1000 Ljubljana, Slovenia; 4 Department of Dermatology, Allergy, and Venereology, University of Lübeck, 23562 Lübeck, Germany; 5 Department of BioMedical Research, University of Bern, 3008 Bern, Switzerland; 6 Department of Rheumatology and Immunology, Inselspital, Bern University Hospital, 3008 Bern, Switzerland

**Keywords:** BET bromodomain protein, I-BET, histone acetylation, chromatin organization

## Abstract

Bromodomain- and extra-terminal domain (BET) proteins are epigenetic reader proteins that regulate transcription of their target genes by binding to acetylated histone side chains. Small molecule inhibitors, such as I-BET151, have anti-inflammatory properties in fibroblast-like synoviocytes (FLS) and in animal models of arthritis. Here, we investigated whether BET inhibition can also affect the levels of histone modifications, a novel mechanism underlying BET protein inhibition. On the one hand, FLSs were treated with I-BET151 (1 µM) for 24 h in absence and presence of TNF. On the other hand, FLSs were washed with PBS after 48 h of I-BET151 treatment, and the effects were measured 5 days after I-BET151 treatment or after an additional 24 h stimulation with TNF (5 d + 24 h). Mass spectrometry analysis indicated that I-BET151 induced profound changes in histone modifications, with a global reduction in acetylation on different histone side chains 5 days after treatment. We confirmed changes on acetylated histone side chains in independent samples by Western blotting. I-BET151 treatment reduced mean TNF-induced levels of total acetylated histone 3 (acH3), H3K18ac, and H3K27ac. In line with these changes, the TNF-induced expression of BET protein target genes was suppressed 5 d after I-BET151 treatment. Our data indicate that BET inhibitors not only prevent the reading of acetylated histones but directly influence overall chromatin organization, in particular after stimulation with TNF.

## 1. Introduction

Acetylation of histone side chains is a key mechanism underlying transcriptional regulation [1]. Bromodomain- and extraterminal domain (BET) proteins bind to ε-N-acetylation modifications via their bromodomains (BRD) and serve as docking platforms for several other regulatory proteins at the chromatin. BET protein family members BRD2, BRD3, BRD4, and the testis-specific BRDT possess two BRDs in tandem arrangement, and hence constitute a unique family among BRD proteins [2].

Synovial fibroblasts (FLS) are the main stromal cells in the joint that maintain joint function and provide a joint-specific environment [3]. Besides immune cells, FLSs carry a substantial genetic risk for the development of rheumatoid arthritis (RA) [4], the most prominent chronic inflammatory joint disease. FLSs are key effector cells that drive joint destruction and inflammation in RA. They have an apoptosis-resistant phenotype and increased levels of proliferation, and they secrete matrix-metalloproteinases (MMP) and inflammatory cytokines and chemokines upon stimulation with cytokines or Toll-like receptor ligands [5]. Recently, FLSs were identified as the culprit cell type underlying tissue priming and mediating a switch from an acute to a chronic joint disease [6]. The activated phenotype of FLSs in RA is concomitant with epigenetic changes, including changes in DNA methylation and in post-translational histone modifications [7]. Histone 3 lysine 27 acetylation (H3K27ac) is an activating histone modification present in active enhancers and promoters [8]. In RA FLSs, a global increase in activating histone marks, in particular levels of H3K27ac, was detected compared to non-RA FLSs [9]. We and others have shown that increased and sustained levels of histone acetylation in inflammatory gene promoters underlie the prolonged chromatin accessibility and sustained transcription of inflammatory genes in FLSs upon applying inflammatory stimuli [10,11]. Together, these studies suggest that interfering with increased levels of histone acetylation is a potential therapeutic strategy in inflammatory joint disease.

Small molecule inhibitors targeting BET proteins were shown to have anti-inflammatory properties in mouse models of arthritis and in different arthritis-relevant cell types, including FLSs [12]. BET inhibitors such as I-BET151 or JQ1 compete for binding to acetylated histone side chains, and displace BRD proteins and mediator complex subunit 1 (Med1) from chromatin [13,14]. BRD4 was shown to assist transcriptional elongation from both enhancers and gene bodies. Thus, BET protein inhibition enables interference with different regulatory elements within the chromatin [15]. In line with this, chromatin immunoprecipitation sequencing experiments in FLSs demonstrated that JQ1 treatment was sufficient to displace BRD2 and BRD4 from chromatin regions overlapping transcription start sites, gene bodies, and enhancers. These changes were associated with a decrease in transcript expression of associated inflammatory genes [16].

So far, all studies in FLSs are based on a short-term treatment of FLSs with BET inhibitors. Here we provide a new concept, in which we treated FLSs with a single dose of I-BET151, followed by a wash-out phase, to monitor long-term effects (5 days) on chromatin modifications and TNF-induced histone modifications.

## 2. Materials and Methods

### 2.1. Patient Samples and Cell Preparation

Synovial tissues were obtained from RA patients undergoing joint replacement surgery (Schulthess Clinic, Zurich, Switzerland). FLSs were isolated and cultured as described elsewhere [3] and used between passages four and eight for all experiments.

### 2.2. Stimulation Experiments

**Figure 1:**
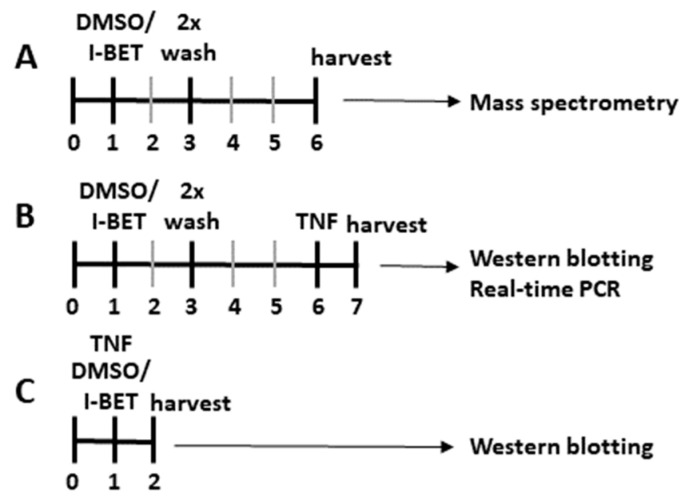
Treatment schemes. (**A**) FLSs were treated with I-BET151 or DMSO (control) on day 1 and (**A**) harvested on day 6 for mass spectrometry, or (**B**) treated with TNF for another 24 h and harvested for Western blotting or RNA isolation. (**C**) FLSs were treated with I-BET151 or DMSO (control) in absence and presence of TNF for 24 h and harvested for Western blotting. In all experiments, control and I-BET151-treated FLSs were derived from the same donors.

FLSs were stimulated with I-BET151 (1 µM, Tocris, Bristol, UK) or matched amounts of DMSO for 48 h. Afterwards, cells were washed with PBS and left untreated for another 72 h (5 d protocol; Figure 1A). FLSs were either harvested for mass spectrometry or treated with TNF (10 ng/µL, R&D systems, Minneapolis, MN, USA) for 24 h and harvested for Western blotting and RNA isolation (Figure 1B). Alternatively, cells were treated with I-BET151 or DMSO in absence and presence of TNF for 24 h (24 h protocol; Figure 1C) and harvested for Western blotting.

### 2.3. Mass Spectrometry

FLSs were treated following the 5 d protocol described above (Figure 1A). FLSs were harvested by trypsinization and washed with PBS. Cell pellets were frozen on dry ice. The relative abundance of >80 post-translational histone modifications was analyzed by mass spectrometry (Mod Spec, Active Motif, *n* = 2/group). In brief, histones were acid-extracted, derivatized via propionylation, and digested with trypsin, then newly formed N-termini were propionylated as previously described [17], and then measured three separate times using a Thermo Scientific TSQ Quantum Ultra mass spectrometer (Waltham, MA, USA) coupled with an UltiMate 3000 Dionex nanoliquid chromatography system (Dionex Corporation, Sunnyvale, CA, USA). The data were quantified using Skyline [18], and represent the percentage of each modification within the total pool of that amino acid residue. To create heatmaps, for each individual modification (including unmodified), the data for each sample were converted to the fraction of the sum across all samples to display relative abundances across the sample group.

### 2.4. Western Blotting

Cells were lysed in Laemmli buffer (62.5 mM TrisHCl, 2% SDS, 10% Glycerol, 0.1% Bromphenolblue, 5 mM β-mercaptoethanol). Whole-cell lysates were separated on SDS polyacrylamide gels and electroblotted onto nitrocellulose membranes (Whatman, Maidstone, UK). Membranes were blocked for 1 h in 5% (*w*/*v*) nonfat milk in TBS-T (20 mM Tris base, 137 mM sodium chloride, 0.1% Tween 20, pH 7.6). After blocking, the membranes were probed with antibodies against histone 3 (H3, dilution 1:5000, #ab1791, Abcam, Cambridge, UK), acetyl-H3 (acH3, dilution 1:500, #06-599, Millipore, Burlington, MA, USA), H3K27ac (dilution 1:600, #ab4729, Abcam), H3K18ac (dilution 1:1000, #39588, active motif), or α-tubulin (dilution 1:10,000, ab7291, Abcam). As secondary antibodies, horseradish peroxidase-conjugated goat anti-rabbit (dilution 1:5000, #111-036-047, Jackson ImmunoResearch, Philadelphia, PA, USA) or goat anti-mouse antibodies (dilution 1:5000, #115-036-062, Jackson ImmunoResearch) were used. Signals were detected using the ECL Western blotting detection reagents (Advansta, San Jose, CA, USA) and the Fusion FX software (Vilber, Sursee, Switzerland).

### 2.5. Real-Time PCR

Total RNA was isolated using the RNeasy Mini Kit (Qiagen), including on-column DNaseI digestion. RNA was reverse-transcribed as described previously [3]. The expression of interleukin (IL) 6, IL8, matrix metalloproteinase (MMP) 1, and MMP3 was measured by real-time PCR using self-designed primers and SYBR green. The expression of ribosomal protein lateral stalk subunit P0 (RPLP0) was measured for sample normalization. Data were analyzed by the comparative Ct method and results are presented as 2^−ΔΔCt^ [19].

### 2.6. Assay for Transposase-Accessible Chromatin Using Sequencing (ATAC-Seq)

ATAC-seq of murine FLSs has been performed previously [20]. In brief, FLSs were cultured from mice that were repeatedly injected with monosodium urate crystals (MSU/MSU). FLSs were treated with I-BET151 (1 µM) or DMSO (control) for 12 h in vitro before harvesting. ATAC-seq libraries were prepared using the ATAC-seq kit from Active Motif, and were sequenced on a HiSeq2500 platform (Illumina, San Diego, CA, USA). Reads were aligned to GRCm38, and MACS2 broad peaks were called. Peaks were annotated relative to gene features using HOMER. Differentially accessible regions were determined using DESeq2 (version 1.28.1).

### 2.7. Statistical Analysis

Statistical analysis on data sets was carried out by using the GraphPad Prism software (San Diego, CA, USA). *N* numbers in all experiments represent biological samples derived from different patients. Differences between experimental groups were analyzed by analysis of variance (ANOVA) followed by Tukey’s multiple comparison test. Data that were not normally distributed were analyzed by Friedman test followed by post hoc Dunn’s multiple comparison test. Data are reported as means ± standard deviations. *p* values < 0.05 were considered significant.

## 3. Results

### 3.1. I-BET151 Induces Global Changes in Post-Translational Histone Modifications

**Figure 2:**
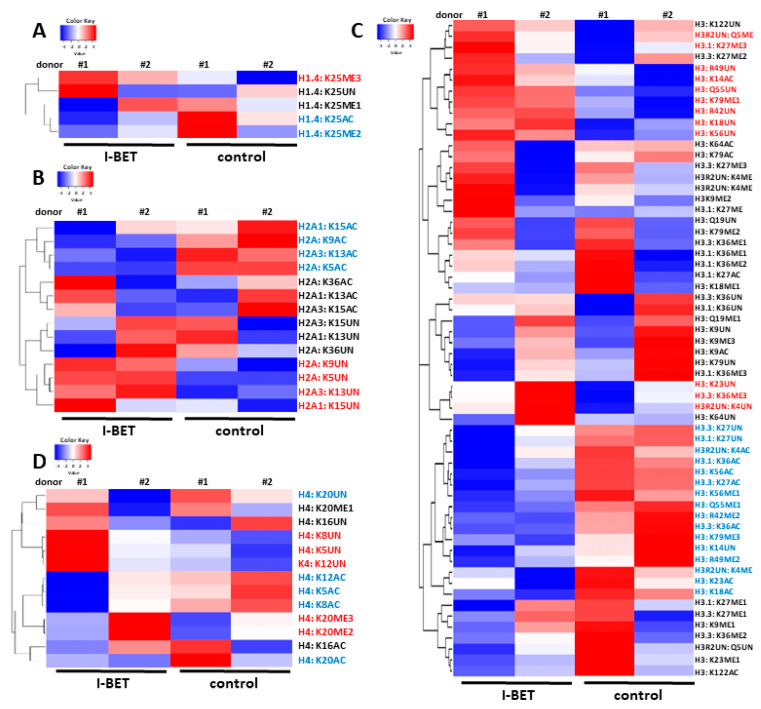
I-BET151-induced changes in FLSs assessed by mass spectrometry. Heatmaps of relative abundancies of histone modifications on (**A**) histone 1 (H1), (**B**) histone 2 (H2), (**C**) histone 3 (H3), and (**D**) histone 4 (H4). I-BET151-induced increases in histone modifications are highlighted in red, the I-BET151-induced suppression of histone modifications is highlighted in blue.

We first performed a mass spectrometry-based screening to assess whether I-BET151 can induce sustained changes on post-translational histone modifications upon a single dose of I-BET151 treatment. We detected I-BET151-induced changes on chromatin modifications on histone 1 (H1; Figure 2A), histone 2 (H2; Figure 2B), histone 3 (H3; Figure 2C), and histone 4 (H4; Figure 2D), with most changes occurring on H3. I-BET151 treatment of FLSs particularly decreased levels of acetylated (AC) lysine (K) residues on different histone proteins, and simultaneously increased levels of unmodified (UN) histone side chains or methylated (ME) histones.

### 3.2. I-BET151 Suppresses TNF-Induced Changes in Histone Acetylation

**Figure 3:**
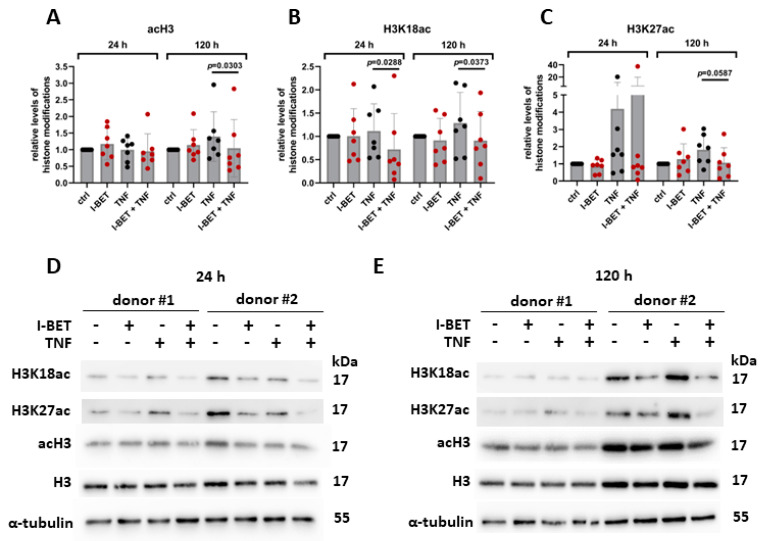
I-BET151-induced changes on TNF-induced histone acetylation in FLSs. FLSs were treated with I-BET151 and TNF for 24 h, following the scheme shown in Figure 1C (24 h). Alternatively, FLSs were pretreated with I-BET151, following the scheme shown in Figure 1B (120 h), followed by a TNF stimulation 24 h prior to harvesting. Densitometric analysis of Western blots (*n* = 7) for (**A**) acetylated histone 3 (acH3), (**B**) H3 lysine 18 acetylation (H3K18ac), (**C**) H3 lysine 27 acetylation (H3K27ac). Changes in histone modifications were quantified relative to the expression of α-tubulin. (**D**,**E**) represent Western blots of results shown in (**A**–**C**). Original membranes are shown in Supplementary Materials.

Since the inflammatory response of FLSs is associated with chromatin reorganization and a de novo writing of activating histone marks [10,11,21], we next verified a potential impact of I-BET151 on the TNF-induced histone acetylation. We either treated FLSs in the absence and presence of TNF for 24 h (outlined in Figure 1C, 24 h) to resemble previously used protocols for assessing I-BET induced effects in FLSs [11,16,22], or alternatively, we pretreated FLSs with I-BET and stimulated FLSs with TNF after 5 days (outlined in Figure 1B, 120 h) to assess histone modifications after 24 h. Pretreatment with I-BET151 for 48 h was sufficient to decrease the levels of total acH3, H3K18ac, and H3K27ac, even after 5 days in TNF-stimulated FLSs. Compared to TNF-stimulated FLSs without I-BET151 pretreatment, levels of acH3 were decreased by 25.2%, (*p* = 0.0303) (Figure 3A); of H3K18ac by 29.3% (*p* = 0.0373) (Figure 3B); and of H3K27ac by 41.7% (*p* = 0.0587) (Figure 3C) in TNF-stimulated FLSs pretreated with I-BET151. A 35.3% (*p* = 0.0288) decrease in H3K18ac levels was additionally observed in FLSs treated with I-BET151 for 24h (Figure 3B), indicating that some I-BET151-induced changes in histone modifications can be already detected after short time exposures.

### 3.3. Long-Lasting Effects of I-BET151 on TNF-Induced Target Genes

**Figure 4:**
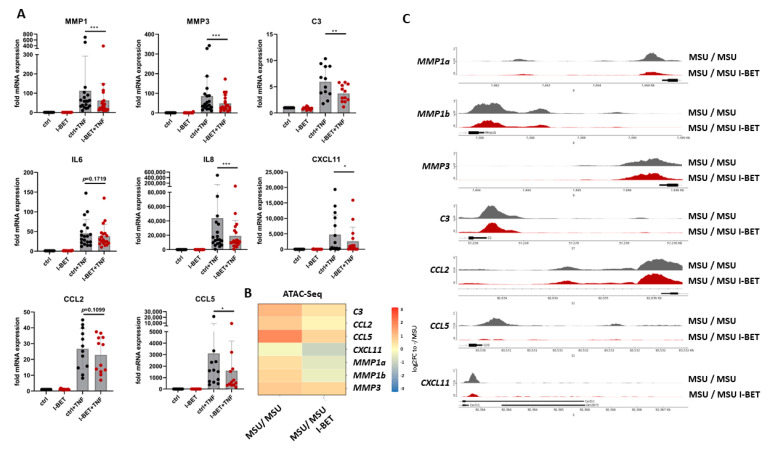
Long-lasting effects of I-BET151 on TNF-induced gene expression and MSU-induced open chromatin states. (**A**) FLSs were treated with a single dose of I-BET151, and treated with TNF after 5 days for 24 h (*n* = 19). The expression of MMP1, MMP3, C3, Il6, IL8, CXCL11, CCL2, and CCL5 was measured by real-time PCR. (**B**) Log2 fold change (FC) in expression of selected genes generated from ATAC-Seq counts on annotated promoters, calculated as the ratio to the mean normalized counts from FLSs isolated from mouse paws after a single injection of MSU crystals (pooled data from 3 replicates from 2 mice/cell pools). (**C**) Genome tracks displaying normalized profiles for ATAC-Seq signals 2 kb upstream of transcription start sites of indicated genes. Each lane shows pooled data from 3 replicates of FLS cultures from 2 mice/pool. * *p* < 0.05, ** *p* < 0.01, *** *p* < 0.001.

Next, we measured the expression of known I-BET151 target genes [20,22] to verify whether long-lasting I-BET151 induced changes on TNF-induced chromatin modifications translate into differences in gene expression. Treatment with I-BET151 suppressed the TNF-induced expression of interleukin 8 (IL8) by 56.1% (*p* < 0.001), matrix metalloproteinase (MMP) 1, and MMP3 by 44.1% (*p* < 0.001) and 42.9% (*p* < 0.001), respectively; C-C motif chemokine ligand (CCL) 5 by 48.1% (*p* < 0.05); and C-X-C motif chemokine ligand (CXCL)11 by 44.6% (*p* < 0.05). Furthermore, we showed a tendency towards a decreased expression of IL6 by 15.5% (*p* = 0.1719) and CCL2 by 14.6% (*p* = 0.1099) (Figure 4A). To assess whether I-BET151 treatment changes the promoter accessibility of these target genes under proinflammatory conditions, we used our existing ATAC-seq data sets derived from murine MSU-primed FLSs (MSU/MSU) that were treated with I-BET151 in vitro [20]. We have previously shown that I-BET151 is sufficient to decrease the expression of a similar set of overlapping target genes in MSU/MSU-exposed murine FLSs and in human FLSs that were repeatedly stimulated with TNF. Our ATAC-seq data indicate reduced levels of DNA accessibility in promoter regions of *C3*, *CCL2*, *CCL5*, *CXCL11*, and *MMP1a*, *MMP1b*, and *MMP3* in I-BET151-treated FLSs (Figure 4B,C).

## 4. Discussion

We and others demonstrated previously that small molecule inhibitors such as I-BET151 or JQ1 suppress the expression of cytokines, chemokines, and MMPs in FLSs after stimulation with a range of inflammatory stimuli, including TNF, IL1, Toll-like receptor ligands, and MSU crystals [11,16,20,22]. Mechanisms underlying these anti-inflammatory effects are still obscure and might involve epigenetic and chromatin-independent mechanisms. Acetylation is a common post-translational modification of histones as well as nonhistone proteins [1]. Accordingly, BET proteins are not only readers of acetylated histone side chains but can also bind to nonhistone proteins, such as acetylated RelA [23]. JQ1 treatment was shown to inhibit the activation of NF-ĸB in FLSs [24]; however, this effect was not observed after treatment of FLSs using I-BET151 [22].

Here, we add a novel mechanism underlying the I-BET151-mediated regulation of gene expression in FLSs. We provide substantial evidence that I-BET151 does not only prevent the reading of acetylated histones but also alters the chromatin organization itself over several days. In vitro histone acetyl transferase assays using recombinant histone proteins revealed that BRD4 has intrinsic histone acetyl-transferase (HAT) activity towards all core histones. In particular, BRD4 was able to acetylate the histone side chains H4K5, H4K8, H4K12, H3K18, H3K27, and H3K122, but not H3K14 and H3K56 [25]. This pattern to a large extent overlaps with histone side chains that consistently possess decreased levels of acetylation in favor of increased levels of unmodified or methylated side chains 5 days after I-BET151 treatment in our mass spectrometry analysis. In contrast, H3K14 was the only side chain that consistently showed increased levels of acetylation, paralleled by a decrease in unmodified H3K14 after I-BET151 treatment. Moreover, in terms of reading, H3K14ac has not been associated with BET proteins but with other members of the BRD protein family, including BRD1 [26], BAZ2A [27], and PBRM1 [28].

In addition to its own HAT activity, an interaction of BRD4 and p300 has been shown to orchestrate the acetylation of H3K27 and H3K56 in pluripotent stem cells. This process was sensitive towards an inhibition with the BET inhibitor JQ1 [29]. Together, these data suggest that I-BET151-mediated effects on chromatin organization might on the one hand reflect direct effects on BET protein HAT activity, and on the other hand reflect indirect effects due to disrupted protein interactions.

BRD4’s HAT activity towards H3K122 has been linked to histone eviction and chromatin opening [25]. We have previously observed that total levels of H3 strongly decreased in promoters of IL6 and IL8 upon stimulation with TNF, indicating that histone eviction is one of the mechanisms underlying the inflammatory response in FLSs [30]. However, global levels of H3 remained stable upon TNF stimulation, and H3 levels in promoters of MMP1 and MMP3 were not altered, indicating that histone eviction is involved in restricted and specific chromatin regions.

Chromatin reorganization, and a persistent acetylation of H3K27 in particular, underlie the inflammatory response of FLSs [11,21]. We have previously shown that the stimulation of FLSs with TNF increased levels of H3K27ac and—at least in samples from some patients—the levels of H3K18ac. This de novo writing of activating histone acetylation marks was to a large extent dependent on the histone acetyl transferase p300 [21]. In addition, a comprehensive comparative analysis of differentially modified epigenetic regions in FLSs derived from patients with RA and osteoarthritis revealed profound differences in histone marks associated with enhancers, such as H3K27ac and H3K4me3, which accounted for the most common differences [9]. Given the efficacy of BET inhibitors to suppress transcription from promoters and enhancers [15], the use of BET inhibitors provides one possibility to interfere with the enhancer activation in FLSs.

A first indication that BET inhibitors might alter the chromatin structure in FLSs globally was observed by Krishna et al., who reported that 24 h treatment with JQ1 reduced levels of open chromatin, measured by ATAC-seq [16]. This effect was in particular evident in FLSs that were stimulated with IL1, and less pronounced in unstimulated cells. This is in line with our own observation that I-BET151-induced effects on histone acetylation at 24 h were only present in TNF-stimulated FLSs. By using mass spectrometry, a more sensitive method than Western blotting, we were additionally able to detect profound changes in I-BET151-induced histone modifications in unstimulated FLSs. We detected a consistent decrease in the acetylation of different histone side chains, in favor of increased levels of unmodified or methylated histone side chains. Given that histone acetylation is an activating histone mark associated with open chromatin and transcriptional activation [31], our data suggest that I-BET151 reduced levels of accessible chromatin in FLSs, and hence suppressed transcription. This is further supported by our own ATAC-seq data sets from murine MSU-primed FLSs. I-BET151 treatment of murine FLSs from MSU/MSU animals decreased the accessibility of promoter regions of genes that were also suppressed by I-BET151 treatment in human TNF-stimulated FLSs over several days. Although our ATAC-seq data sets were obtained from short-term I-BET151 treatments, the changes in chromatin accessibility have relevance in the long-term outcome, as indicated by the corresponding in vivo experiments [20]. In vivo, I-BET151 was sufficient to reverse the MSU-primed transcriptional, metabolic, and pathogenic phenotype of FLSs, leading to a diminished severity of arthritis flares upon reinjection of MSU crystals.

Further evidence for the direct and persistent effects of I-BET151 on histone modifications was provided by a study on the trained immunity of monocytes [32]. In this study, monocytes were treated with I-BET151 prior to induction of trained immunity with β-glucan, followed by a wash-out phase and re-exposure to LPS after 6 days for 24 h. This experimental set up, with a time span between I-BET151 treatment, stimulation of cells, and analysis is almost identical to our own, and led to similar observations. The authors showed that I-BET151 was sufficient to reduce levels of the activating histone marks H3K4me3 and H3K27ac, and to increase the levels of the suppressive H3K9me3 mark in promoter regions of TNF and IL6, concomitant with a suppressed expression of these cytokines in monocytes [32].

## 5. Conclusions

Our data suggest that BET inhibitors do not only prevent the reading of acetylated histone side chains, but also directly affect the chromatin organization, in particular by downregulating global levels of histone acetylation.

## Data Availability

Not applicable.

## Supplementary Materials

The following supporting information can be downloaded at: https://www.mdpi.com/article/10.3390/cells12081149/s1, Figure S1: Original Western blot images for H3K18ac; Figure S2: Original Western blot images for H3K27ac; Figure S3: Original Western blot images for acH3; Figure S4: Original Western blot images for H3; Figure S5: Original Western blot images for α-tubulin.
