# Supplementary material for: Bromodomain Protein Inhibitors Reorganize the Chromatin of Synovial Fibroblasts
Source: Cells. 2023 Apr 13;12(8):1149. doi: 10.3390/cells12081149 (PMC10136646; doi:10.3390/cells12081149)
Supplement: Supplementary file 1 [file cells-12-01149-s001.zip › cells-1958275-supplementary.pdf]

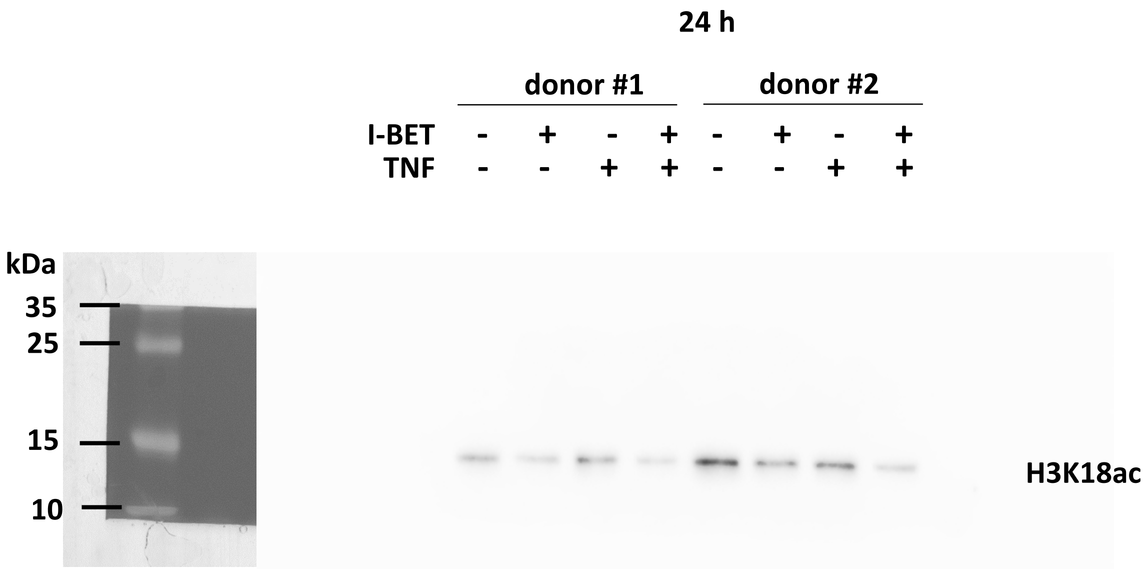

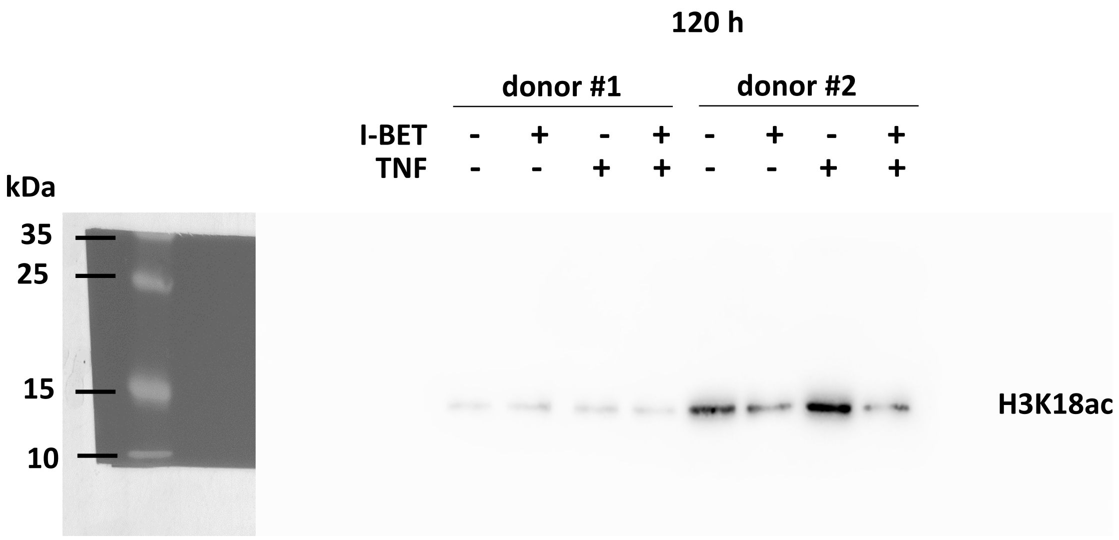

Supplementary Figure S1

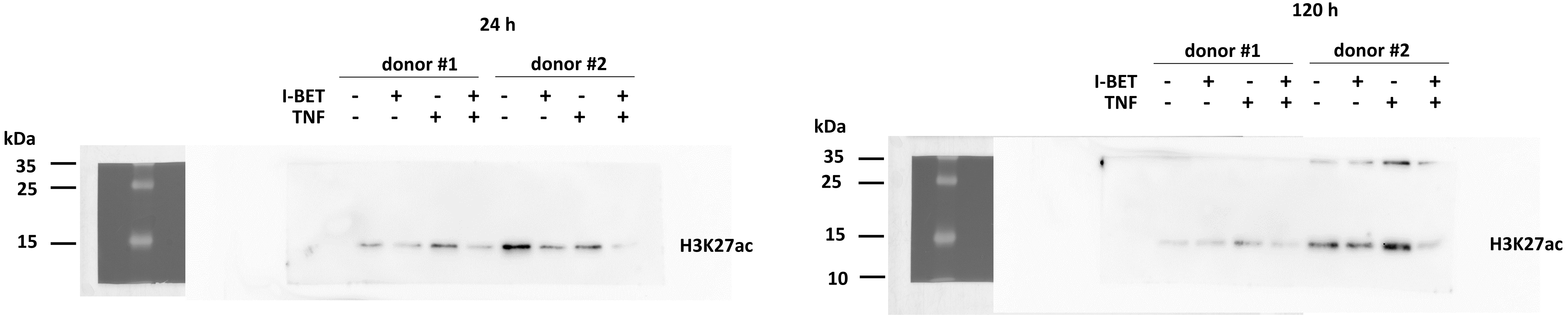

Supplementary Figure S2

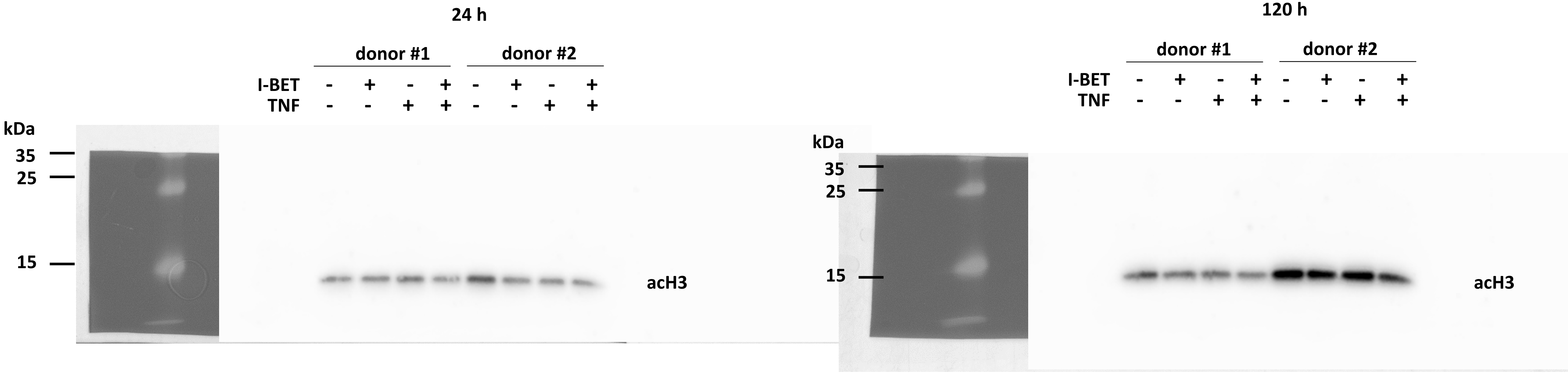

Supplementary Figure S3

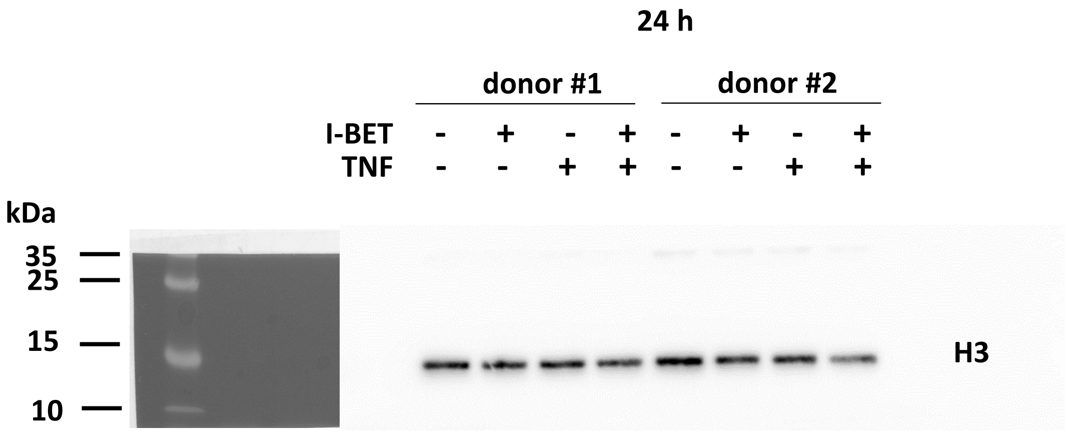

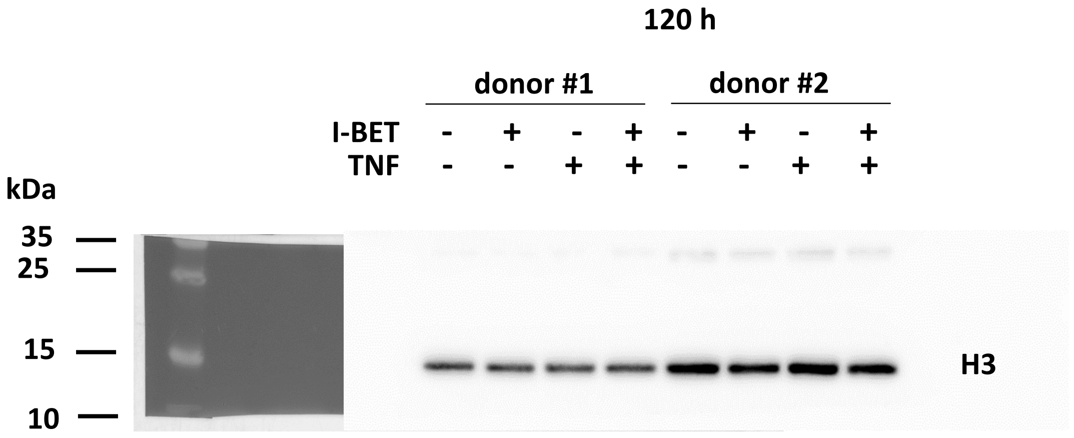

Supplementary Figure S4

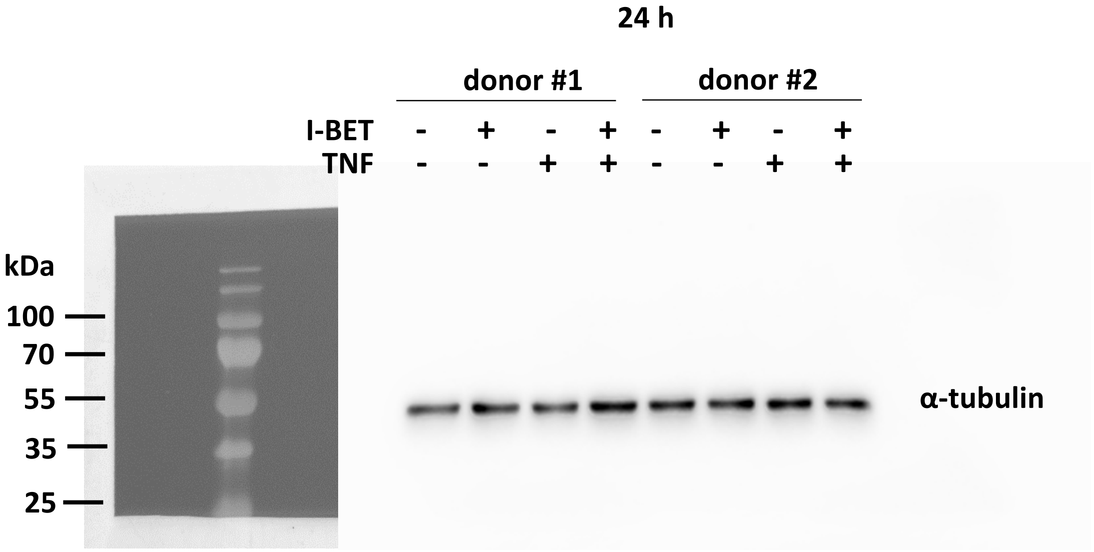

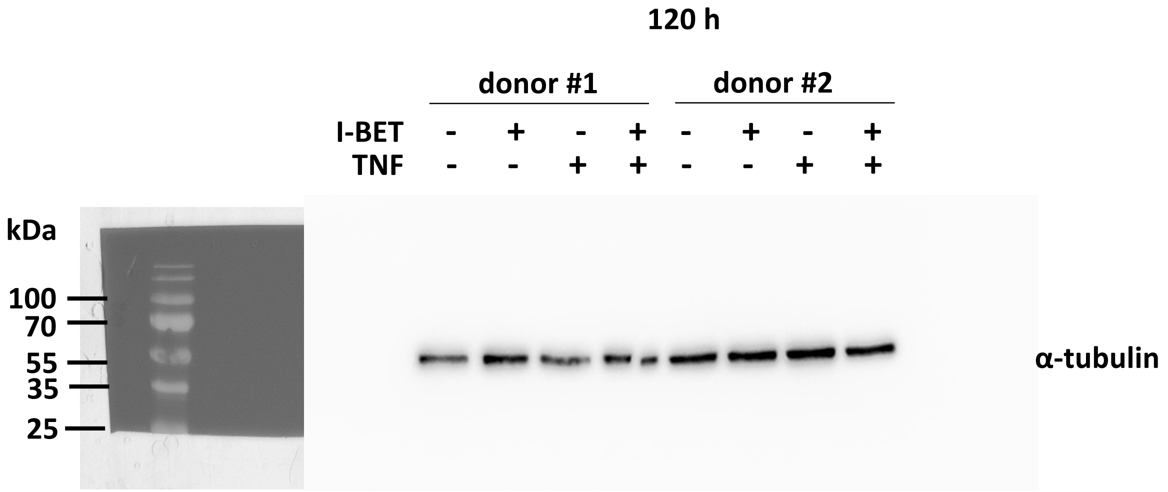

Supplementary Figure S5
